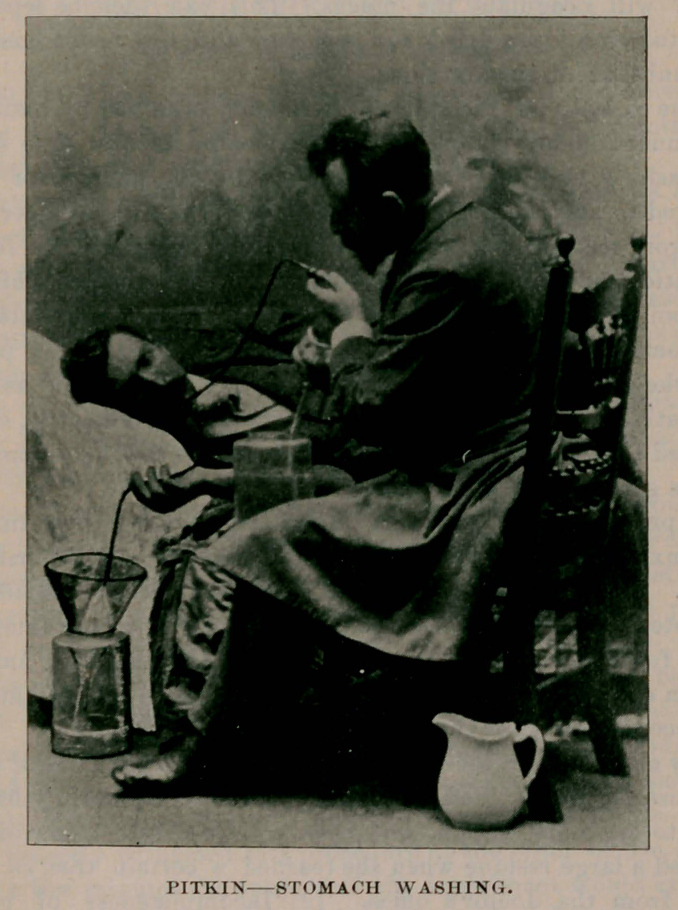# A New Method of Stomach Washing

**Published:** 1895-10

**Authors:** John T. Pitkin

**Affiliations:** Buffalo, N. Y.


### A NEW METHOD OF STOMACH WASHING.

By JOHN T. PITKIN, M. D., Buffalo, N. Y.

WITHIN the past ten years, I have had occasion to practise stomach washing on between two and three hundred private patients, for (*a*) the removal of catarrhal accumulations; (*b*) undigested food; (*c*) poisonous substances; (*d*) foreign bodies; (*e*) the fermentative mass of cholera infantum and chronic dyspepsia; (*f*) as a hepatic stimulant, by converting the stomach temporarily into a hot water bag; (*g*) in atonic dyspepsia, as a stomachic; (*h*) for diagnostic and prognostic purposes; (*i*) to prepare the stomach for forced alimentation, where there is anorexia or repulsion to taking food, *e. g.*, in neurasthenia.

Instead of employing the ordinary stomach-tube with funnel attachment, the objections to which are manifold and to be given later, I resort to a recurrent method of my own, a description of which may prove of interest. Taking two small-sized semi-elastic pieces of white rubber tubing, one yard in length, I introduce them well back into the pharynx of the recumbent patient (dorsal decubitus), *via* mouth or naries. The patient is requested to make frequent efforts to deglutate, intermitting with long inspirations, while the physician gently but persistently presses the tubes onward and downward until one half of their length has been swallowed.

As a preparatory measure, a ten per cent. solution of cocaine muriate, as spray, or dropped in the naries, may be used to allay any reflex irritability of the parts.

Having successfully inserted the stomach-tubes, a common Davidson or bulb syringe should be attached to the distal end of one tube. Then slowly and carefully inject from one to three pints of very warm water (as hot as can be well borne by the hand of the operator), that amount representing about the average normal capacity of the stomach. To introduce more fluid will lead to distressing nausea and vomiting, which is to be avoided, as it discomforts the patient and interferes with the proper cleansing of the viscus, for if once the tubes are ejected and the parts made irritable, the complete success of the procedure will have been jeopardised. When, therefore, two to three pints have been intro-

PITKIN—STOMACH WASHING.

duced, disconnect the syringe from the distal end of the tube, lower the same into a proper receptacle, and the contents of the stomach will be siphoned out. While this is taking place, inject through the second tube more fluid until the stomach discharge consists of only the clear injected water.

Should the tube of egress at any time become clogged, reverse the currents or have the patient add to the vis a tergo by taking a long inspiration. If the mucus accumulations are too viscid to admit of their rapid escape, add liquor calcis to the wash water, one to three, mucine forming a perfect solution with lime water, whereas plain water only holds it in suspension, or forms, at best, a tenacious mixture.

When it becomes desirable to determine the extent of catarrhal disease in a given case, boil the wash water taken from the stomach, which will coagulate the mucus. This can then be separated, collected on a filter, dried and weighed and the result compared with subsequent examinations.

The capacity of the stomach is roughly obtained by noting the maximum amount of injected fluid retainable at any time during the washing, and the degree of acidity by the amount of alkali required to neutralise the same and change the litmus paper reaction from red to blue; lastly, the power of digestion, by feeding the patient who has a cleansed stomach with the chopped white of a hard-boiled egg. Two or three hours later, remove the contents of the stomach, filter the egesta and compare the undigested portion with the amount ingested. More complete methods of analysis, qualitative or quantitative, volumetric and gravometric, can be resorted to in special cases, but, ordinarily, the few simple manipulations given above will suffice.

I prefer the mouth route in willing adults, providing the pharynx is not hypersensitive and the maxillæ and soft parts are intact. With fracture of the jaw, children, hysterical females and intubated patients, the naso-pharynx proves more advantageous, either for cleansing or feeding purposes. By this route, insert a tube in each nostril and proceed as before. Here the patients cannot successfully resist the operator or eject the tubes.

My objections to the established method are: (1) Lack of expelling force, as exemplified by one lady patient complaining that she, much to her mortification, but greatly to her relief, unavoidably vomited a large residue when she reached a certain tree, *en route* home from the doctor's office. (2) Incompleteness of cleansing. After its use, I have immediately employed the recurrent method and found large quantities of mucus and other débris, which it had failed to remove. The residual portion frequently leaves the patient with a sensitive stomach to suffer more or less nausea the remainder of the day. (3) More difficult to perform. I find patients more willing to swallow two small tubes than one which is so much larger. One woman came to me with the complaint that her family physician had endeavored in vain, for over an hour, to pass down her throat his garden hose. (4) Again, limited space absolutely prohibits its successful performance *via* naso-pharynx.

206 CONNECTICUT STREET.

## Figures and Tables

**Figure f1:**